# Diversified *mcr-1*-Harbouring Plasmid Reservoirs Confer Resistance to Colistin in Human Gut Microbiota

**DOI:** 10.1128/mBio.00177-16

**Published:** 2016-04-05

**Authors:** Huiyan Ye, Yihui Li, Zhencui Li, Rongsui Gao, Han Zhang, Ronghui Wen, George F. Gao, Qinghua Hu, Youjun Feng

**Affiliations:** a Department of Medical Microbiology and Parasitology, Zhejiang University School of Medicine, Hangzhou, Zhejiang, China; b Shenzhen Centre for Disease Control and Prevention, Shenzhen City, Guangdong, China; c College of Life Science and Technology, Guangxi University, Nanning City, Guangxi, China; d CAS Key Laboratory of Pathogenic Microbiology and Immunology, Institute of Microbiology, Chinese Academy of Sciences, Beijing, China

## Abstract

Colistin is an ultimate line of refuge against multidrug-resistant Gram-negative pathogens. Very recently, the emergence of plasmid-mediated *mcr-1* colistin resistance has become a great challenge to global public health, raising the possibility that dissemination of the *mcr-1* gene is underestimated and diversified. Here, we report three cases of plasmid-carried MCR-1 colistin resistance in isolates from gut microbiota of diarrhea patients. Structural and functional analyses determined that the colistin resistance is conferred purely by the single *mcr-1* gene. Genetic and sequence mapping revealed that *mcr-1*-harbouring plasmid reservoirs are present in diversity. Together, the data represent the first evidence of diversity in *mcr-1*-harbouring plasmid reservoirs of human gut microbiota.

## IMPORTANCE

The plasmid-mediated mobile colistin resistance gene (*mcr-1*) challenged greatly the conventional idea mentioned above that colistin is an ultimate line of refuge against lethal infections by multidrug-resistant Gram-negative pathogens. It is a possibility that diversified dissemination of the *mcr-1* gene might be greatly underestimated. We report three cases of plasmid-carried MCR-1 colistin resistance in isolates from gut microbiota of diarrhea patients and functionally define the colistin resistance conferred purely by the single *mcr-1* gene. Genetic and sequence mapping revealed unexpected diversity among the *mcr-1*-harbouring plasmid reservoirs of human gut microbiota.

## INTRODUCTION

**FIG 1:**
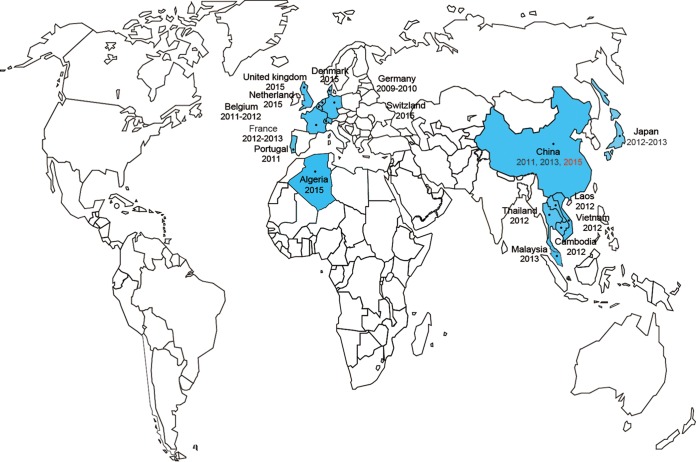
Global distribution of the *mcr-1* colistin resistance gene. The countries where the *mcr-1* gene was discovered are highlighted in blue.

**FIG 2:**
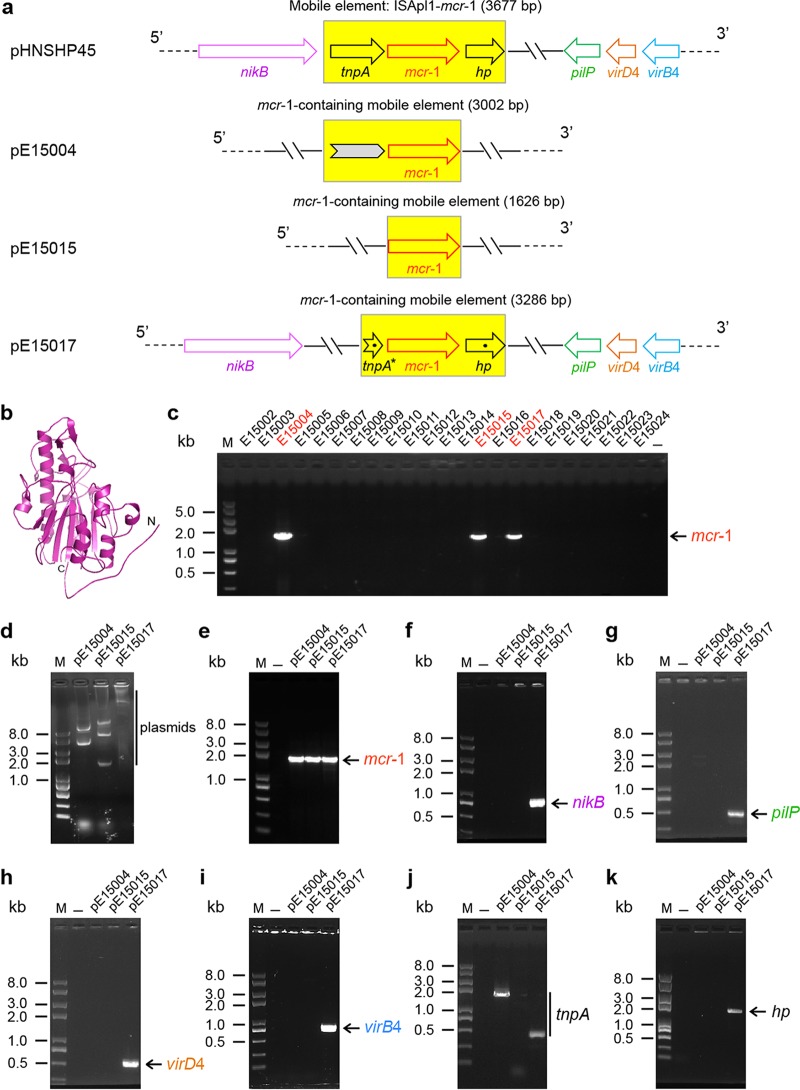
Genetic, structural, and molecular characterization of the *mcr-1*-harbouring plasmids from gut microbiota of diarrhea patients. (a) Scheme for the four *mcr-1*-harbouring plasmids. pHNSHP45 is a plasmid from the Chinese pig isolate of *E. coli* with a known genome sequence (5); the other three plasmids (referred to as pE15004, pE15015,and pE15017) were isolated from three clinical *E. coli* strains (E15004, E15015, and E15017) collected from diarrhea patients admitted to hospitals in Shenzhen City, China, in 2015. Arrows denote the known (and/or putative) genes. A mobile element, ISApl1-*mcr-1* (or an *mcr-1*-containing mobile element), is highlighted with a yellow background. The gray arrow illustrated with plasmid pE15004 (in panel a) represents a DNA fragment of 1,189 bp that shows 100% identity to the equivalent sequence of *Salmonella enterica* serovar Heidelberg plasmid pSH146-32, whereas the *mcr-1* gene of plasmid pE15004 (and/or plasmid pE15015 plus pE15017) is 100% identical to the counterpart gene of *E. coli* SHP45 strain plasmid pHNSHP45 (5). The *mcr-1*-harbouring mobile element candidate from the clinical pE15017 plasmid exhibited 99% identity to that of *E. coli* plasmid pHNSHP45 (5). The broken arrow (tnpA*) denotes the intergenic sequence that is closely next to the *tnpA* gene at the 3-terminus determined in our trials, and the dots indicate that point mutations are present in tnpA* and a hypothetical protein-encoding locus (*hp* gene). PCR-based fine mapping suggested that the pE15017 plasmid carries all four genes (*nikB*, *pilP*, *virD4*, and *virB4*), similarly to *E. coli* plasmid pHNSHP45, but that such is not the case for the other two clinical *mcr-1*-harbouring plasmids (pE15004 andpE15015) that we have reported here. The results validated the idea that the diversified plasmid background is linked to a *mcr-1* colistin resistance gene. (b) Modeled structure of the enzymatic domain of MCR-1 protein. Structural modeling was performed using the program of Swiss model and *Neisseria* lipooligosaccharide phosphoethanolamine transferase A (LptA) as the structural template (PDB: 4KAV), and the resultant output (ribbon photograph) was given using PyMol software. (c) *mcr-1*-based screening of clinical isolates from gut microbiota of diarrhea patients. The expected amplicon of the full-length (~1.6-kb) *mcr-1* gene is indicated with a red arrow, and the clinical *mcr-1*-positive isolates (E15004, E15015, and E15017) are highlighted in red. (d) Profile for plasmids extracted from the clinical *mcr-1*-positive isolates (E15004, E15015, and E15017) determined on the basis of 0.7% agarose gel electrophoresis. (e to i) Molecular detection of the acquired three plasmids using five pairs of specific primers that separately target the *mcr-1* colistin resistance gene (e), *nikB* (f), *pilP* (g), and type IV secretion system-encoding genes *virD4* (h) and *virB4* (i). (j and k) PCR screening for the two neighboring genes of *mcr-1*, the transposase-encoding *tnpA* gene (j) and the hypothetical protein (*hp*)-encoding gene (k). M denotes a Trans2K Plus II DNA ladder (TransGen Biotech, Beijing, China), and minus refers to the negative control.

Colistin (polymyxin E), representing a family of cationic polypeptide antibiotics with broad-spectrum antimicrobial activities, is generally regarded as a last line of refuge (drug/therapeutics) against bacterial infections by the multidrug-resistant Gram-negative pathogens (1, 2). The chromosome-encoded mechanism for colistin resistance in certain members of *Enterobacteriaceae* is associated with two-component systems such as *pmrAB* (3) and *phoPQ* (4) and with the regulator *mgrB* (4), in which the modification of lipid A decreases its affinity to polymyxin (5). Very recently, Liu et al. (5) reported, for the first time, that plasmid-mediated *mcr-1* colistin resistance in animal and human isolates of *Escherichia coli* and *Klebsiella pneumoniae* has emerged in China (Fig. 1 and 2). They defined an unusual mechanism for colistin resistance in that the *mcr-1* gene product belongs to the family of phosphoethanolamine transferase enzymes (Fig. 2b) (5). Our retrospective study showed that the *mcr-1* genes have already been detected in no fewer than 16 countries, including 7 countries in Southeast Asia (China [5, 6], Thailand [7], Laos [7], Japan [8], Vietnam [9], Cambodia [10], and Malaysia [6]) (Fig. 1) and 9 European countries (Denmark [11], United Kingdom [England and Wales] [12], the Netherlands [13], France [7, 12], Portugal [6], Switzerland [14], Germany [15], Belgium [16], and Algeria [7]) (Fig. 1). To the best of our knowledge, the plasmid-borne *mcr-1* gene has been observed in at least 3 enterobacterial species (*E. coli* [5, 11, 13], *Salmonella enterica* [6, 12, 17], and *K. pneumoniae* [5]) and the host reservoirs included at least three kinds of poultry and livestock (chickens [6, 7], pigs [4, 5], and cattle [6]). Of particular note, animal-to-human transmission of MCR-1 colistin resistance has already been found in China (5), Thailand (7), Laos (11), and Denmark (4), raising serious concern about its possible global dissemination and spread (18). So far, it is very true that plasmid pHNSHP45 from the Chinese swine microbiota (Fig. 2a) is the only one (among hundreds of examples of *mcr-1* carriage in animal/human isolates) with the known full genome sequence in China (5).

## RESULTS

### Discovery of three *mcr-1*-positive clinical isolates from human gut microbiota.

**FIG 3:**
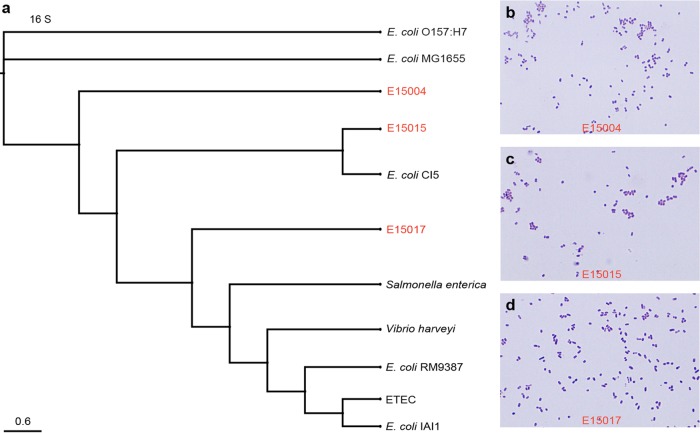
16S-based identification and Gram staining analyses of the three *mcr-1*-harbouring isolates. (a) 16S-based phylogenetic tree of the three *mcr-1*-containing isolates. (b) Gram staining analyses for the E15004 isolate. (c) Gram staining analyses for the E15015 isolate. (d) Gram staining analyses for the E15017 isolate.

Given the fact that current knowledge on the genetic evolution of both the *mcr-1* gene and the *mcr-1*-harbouring vectors and plasmids is extremely limited (and has even lagged), we anticipated that genetic diversity of *mcr-1*-carrying plasmid backbones/reservoirs is probably present in gut microbiota of animals as well as human beings. Here we report that this is the case. We analyzed fecal samples collected from diarrhea patients admitted to hospitals in Shenzhen City, China, in 2015. The patients involved in this study comprised neonates (3 months old), adults (18 to 55 years old), and older persons (83 years old) (not shown). Following routine examination procedures such as 16S sequencing, we investigated 48 isolates of *Escherichia coli* and 27 isolates of *K. pneumoniae* (not shown). PCR screening for the *mcr-1* gene was carried out using a pair of specific primers covering the full length of the *mcr-1* coding sequence (1,626 bp). As a result, 3 of 48 clinical *E. coli* isolates were shown to be *mcr-1* positive in our PCR assays (Fig. 2c), whereas none of the human *K. pneumoniae* isolates were found to be PCR positive for *mcr-1* (not shown). To further confirm the identity of the acquired 3 isolates, 16S-based phylogenetic analyses (Fig. 3a), as well as Gram staining assays, were conducted (Fig. 3b to d). In light of the fact that Liu and coworkers successfully acquired a big plasmid (pHNSHP45; 64,105 bp in length) from a pig *E. coli* isolate (5), we attempted to subject the three *mcr-1*-positive strains (designated E15004, E15015, and E15017 in Fig. 2c) to plasmid isolation by employing manual extraction with the alkaline lysis method. It seems likely that different plasmids are present in those clinical human strains in that their profiles are completely different, at least in 0.7% agarose gel electrophoresis (Fig. 2d). As expected, all three of the acquired plasmids, namely, pE15004, pE15015, and pE15017, contained the *mcr-1* gene, evidenced by our PCR-based determination with these plasmids as the templates (Fig. 2e). Direct DNA sequencing of the three *mcr-1* genes (1,626 bp) that we obtained from human clinical isolates showed that they were 100% identical to all the *mcr-1* genes of diversified origins with known sequences (such as those carried by pHNSHP45 [5] and even the human gut microbiota contig from N009A [6]), indicating that the *mcr-1* colistin resistance gene itself is highly conserved and seems to be under low selective pressure right now.

### *In vivo* evidence for MCR-1-mediated colistin resistance.

**FIG 4:**
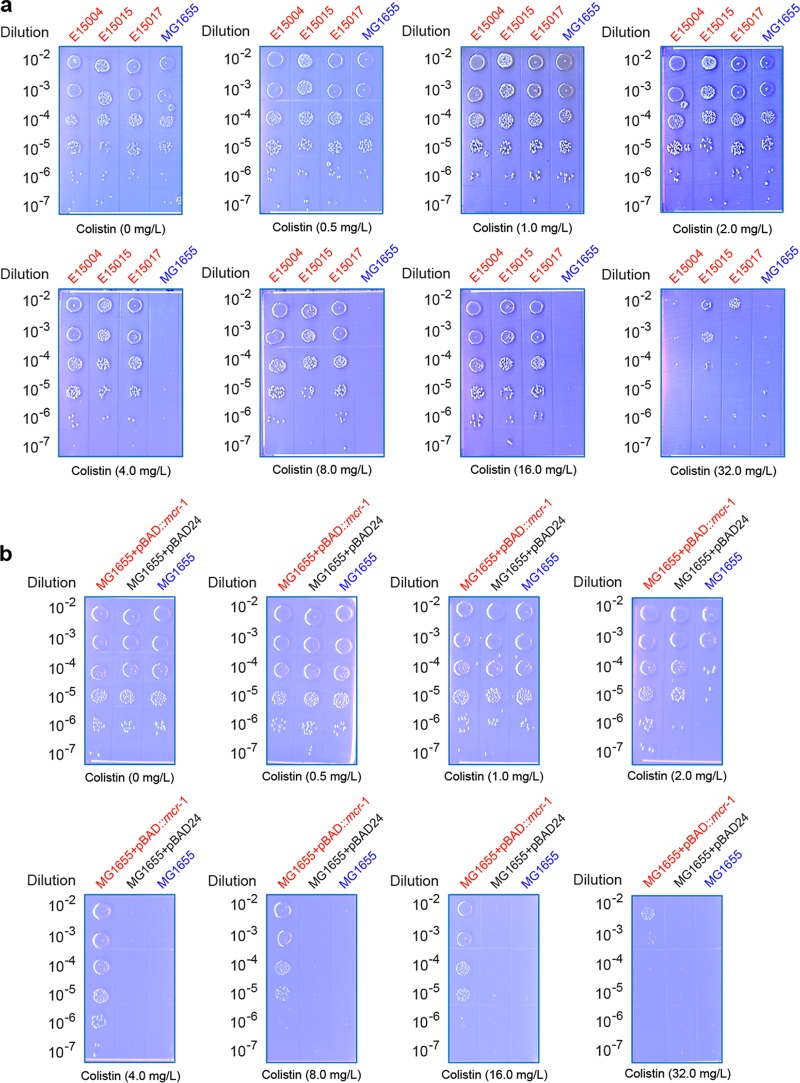
Determination of colistin resistance conferred by the plasmid-borne *mcr-1* gene. (a) Determination of colistin MIC of the clinical *E. coli* isolates from diarrhea patients. (b) Expression of *mcr-1* augments resistance of colistin-susceptible strain *E. coli* MG1655 to the colistin antibiotics. The three clinical *mcr-1*-positive *E. coli* strains (E15004, E15015, and E15017) are highlighted in red, whereas the prototypical wild-type strain (also *mcr-1*-negative strain) of *E. coli*, MG1655, is referred to the negative control (in blue). To determine the MIC of colistin, the mid-log-phase cultures (optical density at 600 nm [OD_600_] = 0.7) in serial dilution were spotted on LBA plates supplemented with colistin at various levels (0, 0.5, 1.0, 2.0, 4.0, 8.0, 16.0, and 32.0 mg/liter) and maintained overnight at 37°C. Expression vector pBAD24 is used for functional cloning of the *mcr-1* gene in *E. coli*. Expression of *mcr-1* is induced by the addition of 0.2% arabinose to LBA media.

Structural modeling of MCR-1 showed that its architecture is similar to that of *Neisseria* lipo-oligosaccharide phosphoethanolamine transferase A (Fig. 2c), implying similar enzymatic mechanisms by which bacterial lipid A modification proceeds. To address the function of *mcr-1 in vivo*, we employed two approaches, one of which was testing the colistin tolerance of the three clinical human *Escherichia* isolates with a natural *mcr-1*-positive plasmid and the other of which was visualizing the effect of colistin-susceptible *E. coli* strain MG1655 by regulated expression of the pure *mcr-1* gene. As we expected, the prototypical strain of *E. coli* MG1655 as the negative control grew on the Luria-Bertani agar (LBA) plates supplemented with no more than 2.0 mg/liter of colistin (Fig. 4a) (note that the resistance of 2.0 mg/liter refers to a diagnostic cutoff/breakpoint [13]). In contrast, the clinical human enteric strains (E15004, E15015, and E15017) consistently exhibited appreciable growth on LBA plates with up to 16 mg/liter of colistin, and the MIC was 32 mg/liter for colistin (Fig. 4a), which is much higher than the 8 mg/liter seen with pHNSHP45 (5). This discrepancy might be in part due to different evaluation methods. When we engineered the *mcr-1* gene into an arabinose-inducible expression vector, pBAD24, for *E. coli*, similar scenarios were observed in our experiments. Although basal expression of *mcr-1* (without the addition of any colistin into growth media) confers resistance of the recipient MG1655 strain to 4 mg/liter colistin on LBA plates, induced expression of *mcr-1* in the presence of 0.2% arabinose increased its colistin tolerance to 16 mg/liter (Fig. 4b). The results showed that all of the *mcr-1* genes in these clinical isolates from gut microbiota of diarrhea patients are functional in colistin resistance.

### Unexpected diversity in *mcr-1*-harbouring plasmid reservoirs.

To further figure out whether or not our plasmids harbour key genetic elements similar to those of pHNSHP45, the only *mcr-1*-harbouring plasmid with a sequenced genome, we designed six more pairs of primers (Table 1) targeting the following six genes (Fig. 2a): first, *nikB* (Fig. 2f) and *pilP* (Fig. 2g) separately encode a relaxase for transposal function and a type IV pilus biogenesis protein; second, *virD4* (Fig. 2h) and *virB4* (Fig. 2i) are two genes encoding two components (VirD4 and VirB4 with an ATPase activity) of a type IV secretion system; third, the two neighboring DNA fragments adjacent to the *mcr-1* gene correspond to the *tnpA* transposase-encoding gene (Fig. 2j) and to a gene encoding a hypothetical protein (*hp*) (Fig. 2k), respectively. To our surprise, PCR assays showed that only the pE15017 plasmid is positive for the first four genes of those six genes (*nikB* [Fig. 2f], *pilP* [Fig. 2g], *virD4* [Fig. 2h], and *virB4* [Fig. 2i]), suggesting that pE15017 probably has high homology to pHNSHP45, whereas the remaining two plasmids (pE15004 and pE15015 [Fig. 2c]) exhibit a different plasmid backbone in that at least those four genes are lacking (Fig. 2f to i). A similar scenario was also noted in the PCR amplification of the *hp* gene adjacent to 3′ end of the *mcr-1* gene (i.e., the pE15017 plasmid is the only *hp* gene-positive one, as shown in Fig. 2k). This speculation was also supported by subsequent direct DNA sequencing of the four genes (*nikB*, *pilP*, *virD4*, and *virB4*) amplified from the pE15017 plasmid in that all of them are 100% identical to the counterparts of pHNSHP45 (Fig. 2a). In contrast, the DNA region covering *tnpA*, another gene that neighbors the *mcr-1* gene, exhibited a differential profile in that the PCR amplicon (~1.5 kb) is present in the pE15004 plasmid, a shortened version of around 0.5 kb (tnpA*) appears in the pE15017 plasmid, but no such band exists in the pE15015 plasmid (Fig. 2j). Further sequence assembly of the DNA fragments overlapping the *mcr-1* gene revealed that unexpected diversity is present the *mcr-1*-harbouring plasmid backbones/reservoirs (Fig. 2a). First, as in the case of the *mcr-1*-containing mobile element (note that only a 3,002-bp sequence is available right now), use of the Basic Local Alignment Search Tool (BLAST) delineated that the sequence upstream of the *mcr-1* gene (1,189 bp) in the pE15004 plasmid is 100% identical to that in *S. enterica* serovar Heidelberg plasmid pSH146-32, whereas the remaining *mcr-1* part completely matches the counterpart of pHNSHP45 (5) (Fig. 2a). Second, the adjacent sequences of *mcr-1* similar to those of pHNSHP45 are absent in the pE15004 plasmid (Fig. 2a). Third, the *mcr-1*-barboring mobile sequence (note that only a 3,286-bp sequence is acquired) in pE15004 showed 99% identity to the equivalent part of pHNSHP45 (5) in that point mutations are present in the shortened version of *tnpA* (namely, tnpA*) and in the *hp* gene (Fig. 2a). Our findings provided direct molecular evidence that the *mcr-1*-carrying plasmid backbones/reservoirs present in the gut microbiota of diarrhea patients are of diversified/hybrid origins, which is somewhat consistent with an *in silico* speculation by Tse and Yuen (17) in a study of *Salmonella* and validates a similar hypothesis by Arcilla et al. (13).

**TABLE 1:** Primers used in this study^a^

Primer	Primer sequence	Target gene (bp)
16S-F	5′ AAATTGAAGAGTTTGATCATGG 3′	16S rRNA gene (1,554)
16S-R	5′ GCTTCTTTAAGGTAAGGAGGT 3′	
*mcr*-1-F	5′ ATGATGCAGCATACTTCTGTG 3′	*mcr-1* (1,626)
*mcr*-1-R	5′ TCAGCGGATGAATGCGGTG 3′	
*nikB*-F	5′ GATGAACTTGATCATCGTGTTGT 3′	*nikB* (705)
*nikB*-R	5′ GTAATTCTGACGAAAAAGAGGA 3′	
*pilP*-F	5′ TTAAAGAATAAGCTGGCGTTTC 3′	*pilP* (495)
*pilP*-R	5′ ATGTTAAAAATAATTAAACCAACG 3′	
*virD*4-F	5′ AATGTCAACATGATTGTTAC 3′	*virD4* (552)
*virD*4-R	5′ GAACATAACCCGGACCTGAAAT 3′	
virB4-F	5′ AACTCTTTTTCAGTAAGCCCAAT 3′	*virB4* (780)
virB4-R	5′ TTAATGTTTGTTGTGGATTACAACC 3′	
*tnpA*-F	5′ GGT TTT CGG GCT TTT TAA GAG 3′	*tnpA* (1,504)
*tnpA*-R	5′ TAG CAC ATA GCG ATA CGA TG 3′	
*hp*-F	5′ GAT AAG CAA ACT GGC ATC ACG 3′	*hp* gene (1,646)
*hp*-R	5′ GAA CCC TGT ATA TAG CCT GTC 3′	

a All of the primers listed originated in this study.

## DISCUSSION

In summary, it is reasonable to surmise that diversified transfer of plasmid-mediated *mcr-1* colistin resistance might be present, and confirmation requires further epidemiological investigations. Given that (i) colistin as a veterinary medicine is extensively applied in agricultural (poultry and livestock) production worldwide, (ii) widespread distribution of the *mcr-1* colistin resistance gene by transposal elements/plasmids occurs in animals, meat/food samples, and human gut microbiota, (iii) the relatively widespread occurrence of *mcr-1* in isolates from no fewer than 10 countries is known right now, and (iv) colistin resistance represents a major breach in our last line of defense against multidrug-resistant bacterial pathogens, it might greatly necessary to clinically monitor the diversified *mcr-1*-harbouring plasmids/reservoirs, reevaluate the efficacy (safety) of colistin in veterinary use, and formulate a comprehensive strategy to fight against plasmid-mediated *mcr-1* colistin resistance, especially in pan-drug-resistant Gram-negative bacterial strains (19).

## MATERIALS AND METHODS

### Bacterial isolations and identification.

Fecal samples were routinely collected from diarrhea patients admitted to hospitals in Shenzhen City, China, in 2015. Luria-Bertani (LB) liquid media (and/or solid agar plates) were applied to isolate the enterobacterial species. To initially determine the bacterial identity, the acquired bacteria were routinely subjected to biochemical tests as well as to colony PCR assays with 16S-specific primers. Gram staining assays were routinely performed. As a result, either *Escherichia coli* or *Klebsiella pneumoniae* was predominantly assigned to the gut microbiota of the diarrhea patients.

### DNA manipulations.

To probe the presence of the *mcr-1* gene, all of the bacterial isolates harvested (75 in total [48 for *E. coli* and 27 for *K. pneumoniae*]) were screened via PCR-based diagnostics with *mcr-1*-specific primers (Table 1). The *mcr-1*-positive bacteria were further subjected to plasmid isolations with the manual alkaline lysis method. Then, the plasmid-harbouring isolates were dissected finely using multiplex PCR with six pairs of specific primers targeting six different loci (such as *virD4* and *virB4*, encoding a type IV secretion system) (Table 1). All of the PCR-amplified DNA fragments were verified by direct DNA sequencing and analyzed by ClustalW-aided multiple-sequence alignments. The *mcr-1* gene was cloned into the arabinose-inducible expression vector pBAD24 in *E. coli* (20), giving the recombinant plasmid pBAD24::*mcr-1*.

### Determination of colistin MIC.

Mid-log-phase bacterial cultures in a dilution series were spotted on LBA plates supplemented with various levels of colistin (0, 0.5, 1.0, 2.0, 4.0, 8.0, 16.0, and 32.0 mg/liter) and kept at 37°C overnight. In the assays of inhibitory colistin concentrations, the findings for all three of the *mcr-1*-containing strains (E15004, E15015, and E15017) were confirmed, whereas the negative control corresponded to MG1655, the prototypical strain of *E. coli*. To further examine the function of the single *mcr-1* gene *in vivo*, we monitored the resistance of the colistin-susceptible MG1655 strain conferred by expression of arabinose-inducible, pBAD24::*mcr-1*-borne *mcr-1*.

### Bioinformatics analyses.

The acquired 16S sequences in full length were subjected to phylogenetic analyses using the webserver of Clustal Omega. The coding sequences of the *mcr-1* gene and the other target genes such as *virD4* and *virB4* were aligned with their counterparts with known sequences using BLAST.
